# Na,K-ATPase Kinetics and Oxidative Stress in Kidneys of Zucker Diabetic Fatty (fa/fa) Rats Depending on the Diabetes Severity—Comparison with Lean (fa/+) and Wistar Rats

**DOI:** 10.3390/biology11101519

**Published:** 2022-10-17

**Authors:** Norbert Vrbjar, Tomas Jasenovec, Marta Kollarova, Denisa Snurikova, Maria Chomova, Dominika Radosinska, Ivana Shawkatova, Lubomira Tothova, Jana Radosinska

**Affiliations:** 1 Centre of Experimental Medicine, Slovak Academy of Sciences, Institute for Heart Research, Dúbravská Cesta 9, 841 04 Bratislava, Slovakia; 2 Institute of Physiology, Faculty of Medicine, Comenius University in Bratislava, Sasinkova 2, 811 08 Bratislava, Slovakia; 3 Premedix Academy, Medená 18, 811 02 Bratislava, Slovakia; 4 Institute of Medical Chemistry, Biochemistry and Clinical Biochemistry, Faculty of Medicine, Comenius University in Bratislava, 811 08 Bratislava, Slovakia; 5 Institute of Immunology, Faculty of Medicine, Comenius University in Bratislava, 811 08 Bratislava, Slovakia; 6 Institute of Molecular Biomedicine, Faculty of Medicine, Comenius University in Bratislava, 811 08 Bratislava, Slovakia

**Keywords:** type 2 diabetes mellitus, kidney, ZDF rats, fa/fa, fa/+, sodium pump, carbonyl stress, antioxidant state

## Abstract

**Simple Summary:**

We investigated sodium–potassium pump (Na,K-ATPase) activity and oxidative stress markers in kidney samples of obese Zucker diabetic fatty (ZDF) rats to characterize this animal model of type 2 diabetes mellitus (T2DM) more thoroughly. Two controls—lean ZDF counterparts and Wistar rats—were used. We hypothesized that renal parameters depend on T2DM severity, as well as on the applied control. Our results suggest that the similar genetic background of obese and lean ZDF rats makes lean ZDF controls predisposed to abnormalities observed in obese counterparts. Obese ZDF rats with well-developed T2DM showed higher lipid peroxidation in the renal medulla and a higher ability of Na,K-ATPase to utilize the energy substrate in comparison with obese ZDF rats with lower glycemia. In comparison with both controls, Na,K-ATPase enzyme activity was higher in the renal cortex of ZDF rats independent of diabetes severity. This might be a consequence of increased glucose load in tubular fluid, as the transport of Na^+^ from the tubular cells is necessary for glucose reabsorption. PubMed database revealed more than 6000 results (August 2022) for the keyword “Zucker rat”, confirming their intense usage in research; therefore, a comprehensive characterization of this T2DM model is desirable.

**Abstract:**

For a better insight into relations between type 2 diabetes mellitus (T2DM) and Na,K-ATPase properties in kidneys, we aimed to characterize two subgroups of ZDF obese (fa/fa) rats, with more and less developed T2DM, and compare them with two controls: lean (fa/+) and Wistar. Na,K-ATPase enzyme kinetics were estimated by measuring the ATP hydrolysis in the range of NaCl and ATP levels. As Na,K-ATPase is sensitive to oxidative stress, we evaluated selected oxidative stress parameters in kidney homogenates. Our results suggest that thiol–disulfide redox balance in the renal medulla and Na,K-ATPase properties in the renal cortex differ between both controls, while observed measurements in lean (fa/+) rats showed deviation towards the values observed in ZDF (fa/fa) rats. In comparison with both controls, Na,K-ATPase enzyme activity was higher in the renal cortex of ZDF rats independent of diabetes severity. This might be a consequence of increased glucose load in tubular fluid. The increase in lipid peroxidation observed in the renal cortex of ZDF rats was not associated with Na,K-ATPase activity impairment. Regarding the differences between subgroups of ZDF animals, well-developed T2DM (glycemia higher than 10 mmol/L) was associated with a higher ability of Na,K-ATPase to utilize the ATP energy substrate.

## 1. Introduction

On the cellular level, the first site where cells respond to various extracellular impulses is the plasma membrane. Among the most important impulses modifying the enzymatic and transport functions of integrated plasma proteins appertain the impulses which influence the transmembrane ion transport. In maintaining intracellular homeostasis of monovalent ions, a crucial role is ascribed to Na,K-ATPase that transforms the chemical energy of ATP to preserve electrochemical sodium and potassium gradients across membranes of all living cells in the organism. The functional status of Na,K-ATPase in kidneys is extremely important, as the enzyme contributes to the maintenance of multiple transport mechanisms responsible for the passage of substances across the tubular cells and, in particular, the proper homeostasis of sodium in the organism. Properties of the Na,K-ATPase enzyme are very sensitive to various physiological and pathophysiological conditions, including hypertension or diabetes.

Previous studies showed that renal Na,K-ATPase reacts in a different way to complications induced by the two most common types of diabetes mellitus, type 1 and type 2. The insulin-dependent type 1 diabetes mellitus is associated with lowered expression of the enzyme as well as decreased activity in kidneys, as was revealed in various studies using the model of streptozotocin-induced diabetes in experimental animals [1,2,3,4,5,6]. However, Na,K-ATPase activity was shown to be unchanged in proximal tubules of young rats suffering from non-insulin-dependent type 2 diabetes mellitus (T2DM) [7]. For investigation of renal Na,K-ATPase properties in the condition of T2DM, the strain of Zucker diabetic fatty (ZDF) rats represents an important source of information among various animal models used. ZDF rats are characterized by hyperphagia and obesity leading to diabetes mellitus due to a missense mutation in the gene coding the leptin receptor (fa/fa). For a better insight into relations between T2DM and Na,K-ATPase properties in kidneys, we aimed to compare two groups of older ZDF rats- with more and less developed T2DM. Assigning the ZDF rats according to glycemia might be desirable, as suggestions have been made that variations in ZDF rat characteristics occurred in aged animals [8,9,10]. In the majority of studies, ZDF rats are compared with Zucker lean controls (fa/+). Since they have a similar genetic background to ZDF animals, Zucker lean rats may exhibit certain types of renal pathology in comparison with wild-type rats. Therefore, the second aim of the study was to observe possible differences in renal Na,K-ATPase kinetics between two age-matched controls—the commonly used fa/+ lean rats and the independent control Wistar rats.

Oxidative stress was recognized as a significant contributor to the development of T2DM complications [11], including renal complications. The oxidative imbalance in the renal tissue of obese and diabetic Zucker rats was documented in previously published studies showing an increased presence of malondialdehyde, decreased levels of reduced glutathione (GSH), and decreased superoxide dismutase activity [12,13,14,15,16]. It is worth noting that the Na,K-ATPase enzyme represents one of the cellular targets of oxidative stress. Proximal tubular Na,K-ATPase enzyme activity was shown to be sensitive to oxidative modification [17]. It was suggested that the most significant consequence of oxidative injury is impaired Na,K-ATPase activity as well as the inability of Na,K-ATPase molecules to form oligomers and, in this way, interact with each other [18]. Taking this into consideration, we also aimed to monitor selected parameters reflecting the oxidative stress status in the renal tissue of all experimental animals.

## 2. Materials and Methods

### 2.1. Animal Model

The following groups of male laboratory rats were investigated in the study: Wistar rats (further referred to as CW, *n* = 8) serving as an independent control, ZDF lean fa/+ rats (further referred to as ZL, *n* = 8) serving as a standard and commonly used control for ZDF fa/fa rats, and ZDF fa/fa rats (*n* = 16). The ZDF fa/fa group was further arbitrarily divided into two subgroups according to fasting glycemia measured at 36 weeks of age: ZDF rats with well-developed hyperglycemia—higher than 10 mmol·L^−1^ (ZDF fa/fa obese diabetic, further referred to as ZOD, *n* = 8), and ZDF rats with lower glycemia—below 10 mmol·L^−1^ (ZDF fa/fa obese, further referred to as ZO, *n* = 8). During the experiment, all animals were housed under a 12 h light—12 h dark cycle, at a constant temperature (20–22 °C) with water and food ad libitum (3rd–7th week of life on a standard pellet diet, from 8th week on Purina Rodent LabDiet 5008, LabDiet, London, UK; components in %: protein 23.5, fat—ether extract 6.5, fat—acid hydrolysis 7.5, fiber 3.8, ash 6.8; calories provided by: protein 26.85%, fat 16.71%, carbohydrates 56.44%). A detailed description of the overall experimental design (including the genotyping of the rats) as well as changes in body weight, non-fasting concentration of blood glucose, and plasma insulin levels during the experiment were provided previously [10]. Glucose and insulin levels were determined in all rats at 36 weeks of age following 16 h of fasting. Blood was taken from the tail under sevoflurane anesthesia. Blood glucose level was determined by a glucometer (FreeStyle Optium, Abbott, Maidenhead, UK) and plasma insulin using the Rat/Mouse Insulin ELISA Kit (EZRMI-13K, Merck-Millipore, Darmstadt, Germany). At the end of the experiment, between the 38th–39th week of life, the rats were placed in an anesthetic chamber to be exposed to sevoflurane anesthesia at 2.5% inspired fraction until the loss of righting reflex was achieved. Afterwards, euthanasia was performed by decapitation. Both kidneys were rapidly excised and weighed. The left kidney was immediately macroscopically dissected on an ice plate to separate the cortical and medullary part for the determination of oxidative stress parameters. The cortical tissue was used to prepare the plasma membrane fraction for measurements of Na,K-ATPase enzyme kinetics. Though the amount of tissue was sufficient to determine oxidative stress parameters in both parts of the kidney—cortex and medulla—the evaluation of Na,K-ATPase enzyme kinetics requires more material. Therefore, Na,K-ATPase activity was measured only in the renal cortex. After preparation, all samples were rapidly frozen in liquid nitrogen and stored at –80 °C until the analyses.

To assess the kidney function, plasma urea concentration was determined in blood plasma by the kinetic method using the Randox Urea kit (Randox Laboratories Ltd., Crumlin, UK). For this analysis, blood was collected in K_3_EDTA tubes (S-Monovette^®^ K3; Sarstedt, Nümbrecht, Germany) following decapitation.

All experimental animals were imported from the breeding facility at the Department of Toxicology and Laboratory Animal Breeding, Centre of Experimental Medicine, Slovak Academy of Sciences, Dobra Voda, Slovak Republic. The experimental procedures were approved by the Department of Animal Wellness, State Veterinary, and Food Administration of the Slovak Republic (approval number: Ro-493/18-221/3) in accordance with the European Convention for the Protection of Vertebrate Animals used for Experimental and other Scientific Purposes, Directive 2010/63/EU of the European Parliament.

### 2.2. Characterization of Oxidative Status and Consequences of Increased Glycemia

Indicators of oxidative stress, antioxidant capacity, and carbonyl damage were analyzed in both the cortex and the renal medulla in 10% tissue homogenate in phosphate-buffered saline. As a general marker of oxidative stress, the ratio of reduced and oxidized glutathione (GSH/GSSG) representing the thiol–disulfide redox balance was examined. The oxidation of proteins was estimated via measurements of advanced oxidation protein products (AOPP) and for lipid peroxidation, the measurement of thiobarbituric acid-reacting substances (TBARS) was utilized. The antioxidant status was evaluated by measuring the total antioxidant capacity (TAC) and ferric reducing antioxidant power (FRAP). The degree of carbonyl damage was determined based on the concentration of advanced glycation end products (AGEs) and fructosamine. A bicinchoninic Acid Kit was used to determine the protein concentration according to the manufacturer’s instructions. The Synergy H1 Hybrid Multi-mode multi-detection microplate reader (Agilent, Santa Clara, CA, USA) was used for spectrophotometric and fluorometric measurements, while all reagents were obtained from Sigma-Aldrich (Steinheim, Germany). All the above methods were described in detail previously [19].

### 2.3. Plasma Membrane Fraction Preparation

To isolate the plasma membrane fraction of renal tissue, we proceeded according to Jorgensen with slight modifications [20]. Briefly, the renal cortex was homogenized in a cold medium consisting of 250 mmol·L^−1^ sucrose, 25 mmol·L^−1^ TRIS, and 1 mmol·L^−1^ EDTA (pH 7.4) using a tissue homogenizer. The homogenate was centrifuged at 6000× *g* for 15 min. The pellet was homogenized and centrifuged again. The supernatants from both centrifugations were centrifuged at 48,000× *g* for 30 min, and the resulting pellet was suspended in the isolation medium. The protein concentration was determined according to the method of Lowry et al. [21], and bovine serum albumin was used as a standard. Due to the necessity of having enough material for completing the measurements of enzyme kinetics, the samples of the renal cortex were investigated. Thus, Na,K-ATPase enzyme kinetics could be attributed mainly to proximal and distal tubules of the kidney.

### 2.4. Na,K-ATPase Enzyme Kinetics

Na,K-ATPase sodium-binding properties were estimated by measuring the ATP hydrolysis by 10 μg proteins from plasma membrane fraction in the range of NaCl 2–100 mmol·L^−1^ at 37 °C in the final reaction volume of 0.5 mL. Samples were diluted in a reaction buffer consisting of 50 mmol·L^−1^ TRIS (pH 7.4), 4 mmol·L^−1^ MgCl_2_, and 10 mmol·L^−1^ KCl. After a 20 min preincubation in the absence of substrate, the addition of ATP (8 mmol·L^−1^, Sigma-Aldrich, Steinheim, Germany) initiated the reaction. After 20 min, it was stopped by the addition of 0.6 mL ice-cold solution of 12% trichloroacetic acid. The inorganic phosphorus that was liberated during the reaction was determined according to Taussky and Shorr [22]. The difference between the activities measured in the presence of Na^+^, K^+^, and Mg^2+^ ions and the activity in the presence of only Mg^2+^ ions is related to Na,K-ATPase enzyme activity.

A similar approach was used to estimate ATP utilization by Na,K-ATPase. The concentration of ATP varied (0.16–8.0 mmol·L^−1^), while NaCl concentration was constant (100 mmol·L^−1^).

All reagents for plasma membrane fraction preparation and Na,K-ATPase enzyme kinetics were obtained from Sigma-Aldrich (Steinheim, Germany).

Finally, kinetic parameters V_max_, K_m_, and K_Na_ were evaluated from the data by direct nonlinear regression according to the Michaelis–Menten equation. The V_max_ parameter corresponds to the maximal velocity of enzyme reaction. The K_m_ and K_Na_ values correspond to the concentrations of substrate ATP and cofactor Na^+^ necessary for half-maximal enzyme activation.

### 2.5. Statistical Analysis

The data are presented as means ± standard errors of mean, while their normality was checked using the D’Agostino–Pearson test. One-way analysis of variance followed by Tukey’s post hoc test was used to identify the differences between the groups. For statistical calculations and graphical illustrations, GraphPad Prism 7 and SigmaPlot 13 statistical software were used.

## 3. Results

### 3.1. General Characteristics of the Experimental Animals

General parameters such as body and kidney weight, blood glucose, and plasma insulin concentration are presented in Table 1.

The ZL group showed lower body weight (BW) by 16%, whereas the ZO group revealed higher BW by 27% in comparison with Wistar rats, which served as an independent control. Comparison with the ZL group showed higher BW by 51% and by 27% in the ZO and ZOD groups, respectively. Compared with the ZO group, the BW was lower by 16% in the ZOD group.

A similar trend was observed regarding kidney weight (KW). Comparison with the CW group showed lower KW by 15% in the ZL animals, while it was higher by 10% and by 22% in the ZO and ZOD groups, respectively. Compared with the ZL group, rats in ZO and ZOD groups showed higher KW by 29% and 44%, respectively. Compared with the ZO group, the KW was higher by 11% in the ZOD group. However, the relative kidney weight represented by the KW/BW ratio was markedly higher in the ZOD group in comparison with all other groups. In the ZO group, the KW/BW ratio was lower than in ZL rats.

The fasting blood glucose in samples collected 2 weeks before terminating the experiment was similar in CW and ZL groups. In the ZO group, the glucose level was higher by 31% and in the ZOD group by 173% when compared with the ZL group. Blood glucose level was approximately twice as high in the ZOD group than in the ZO group.

Simultaneous measurements of plasma concentration of insulin showed the following differences in comparison with the CW group: 80% lower levels in ZL, 144% higher levels in ZO, and no significant change in ZOD animals. Comparison with the ZL group yielded a 12 times higher concentration of insulin in the ZO group, while in the ZOD group, this parameter was only 5.4 times higher. A direct comparison of the ZOD group with the ZO group showed a 55% lower insulin level in ZOD.

In comparison with the CW group, plasma urea concentration was higher in ZL (in mmol/L: 4.4 ± 0.3 in ZL vs. 3.2 ± 0.1 in CW, *p* = 0.0068) and ZOD (in mmol/L: 6.3 ± 0.2 in ZOD vs. 3.2 ± 0.1 in CW, *p* < 0.0001) groups. The difference between the ZL and ZOD groups was statistically significant, as well (in mmol/L: 4.4 ± 0.3 in ZL vs. 6.3 ± 0.2 in ZOD vs. 3.2 ± 0.1, *p* = 0.0001). Within ZDF (fa/fa) rats, plasma urea levels were higher in ZOD than in ZO rats (in mmol/L: 6.3 ± 0.2 in ZOD vs. 4.0 ± 0.4 in ZO, *p* < 0.0001). The levels of urea in blood plasma observed in the ZO group showed no differences when compared with CW (in mmol/L: 4.0 ± 0.4 in ZO vs. 3.2 ± 0.1 in CW, *p* = 0.28) and ZL (in mmol/L: 4.0 ± 0.4 in ZO vs. 4.4 ± 0.3 in ZL, *p* = 0.70) groups.

### 3.2. Oxidative Status and Consequences of Increased Glycemia

Measurements of the general indicator of oxidative stress, the GSH/GSSG ratio, showed slight localization-dependent differences in the renal tissue. While, in the cortex, the value of this parameter was similar in all four investigated groups, in the medulla, Wistar rats showed a tendency for a lower GSH/GSSG ratio when compared with all other groups—ZL, ZO, and ZOD. However, only the ZL group differed significantly (Table 2). The status of proteins estimated via measurements of AOPP did not differ significantly in either kidney region (Table 2). On the other hand, lipid peroxidation measured by TBARS was higher in the cortex of both ZDF groups (ZO and ZOD) when compared with control CW and ZL rats (Table 2). In the cortex, the TAC was similar in both control groups, i.e., in CW and ZL rats. A statistically significant increase in TAC was observed in both groups of ZDF rats, regardless of the severity of diabetes mellitus (Table 2). In the medulla, the TAC parameter revealed similar values in all four experimental groups. The value of the second parameter of antioxidant status, the FRAP, was similar in all experimental groups independent of the localization in the renal tissue (Table 2).

The degree of carbonyl damage determined by fructosamine concentration showed similar values in all four experimental groups in the cortex as well as in the medulla of kidneys (Table 3). Measurements of the second parameter of carbonyl damage, the AGEs, showed higher concentrations in the cortex of ZL rats when compared with the ZO group. However, the differences in the level of AGEs were not statistically significant in the renal medulla (Table 3).

### 3.3. Na,K-ATPase

**Figure 1:**
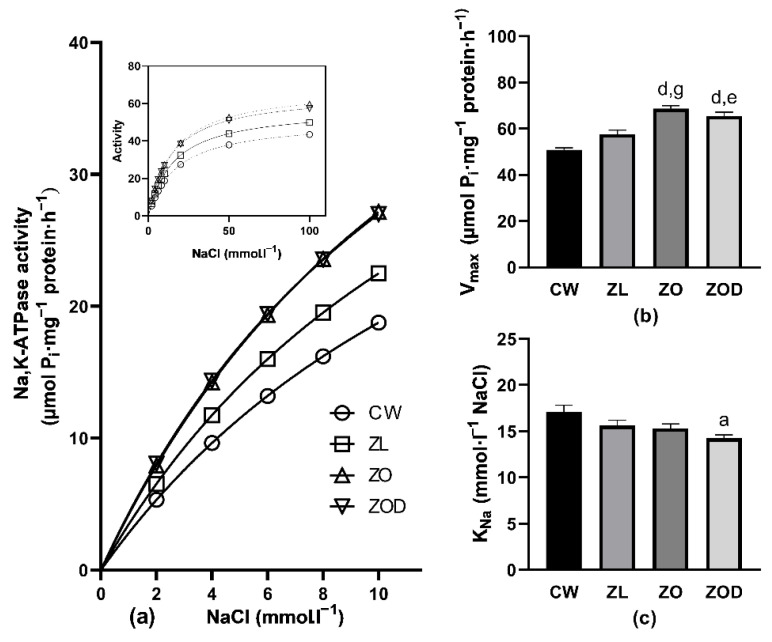
Activation of the Na,K-ATPase enzyme by cofactor Na^+^ in the renal cortex. (Panel **a**) Activation of the Na,K-ATPase by low concentrations of cofactor Na+. Inset: activation of the enzyme in the whole investigated concentration range of Na^+^ in Wistar rats (CW, *n* = 8), lean fa/+ (ZL, *n* = 8), and Zucker diabetic fatty (ZDF) fa/fa rats divided into ZO (ZDF fa/fa obese, glycemia < 10 mmol·L^−1^, *n* = 8) and ZOD (ZDF fa/fa obese diabetic, glycemia > 10 mmol·L^−1^, *n* = 8) groups. Error bars are not depicted, as they are shorter than the size of the symbols. (Panel **b**) V_max_ values in all experimental groups. (Panel **c**) K_Na_ values in all experimental groups. The animals were sacrificed at the age of 38–39 weeks. Data represent means ± standard errors of mean. For statistical analyses, one-way analysis of variance with Tukey’s post hoc test was used: a: *p* < 0.05, b: *p* < 0.01, d: *p* < 0.0001 vs. CW; e: *p* < 0.05, g: *p* < 0.001 vs. ZL.

Activation of the renal Na,K-ATPase with increasing NaCl concentration revealed higher activities in all groups of Zucker rats (ZL, ZO, ZOD), throughout the investigated concentration range (Figure 1a). A comparison of the ZL group with the CW group showed the greatest difference in enzyme activity in the presence of 2 mmol∙L^−1^ NaCl, reaching 23%. With increasing NaCl concentration, the difference in activity gradually decreased to 15% when observed in the presence of 100 mmol∙L^−1^ of NaCl. Evaluation of the above data by the method of nonlinear regression resulted in a significant increase in V_max_ by 13%, but the K_Na_ value remained unchanged (Figure 1b,c). In the ZO group, the difference in the enzyme activity was even higher, ranging from 49% to 37%, observed in the above-described concentration range of NaCl (Figure 1a). Evaluation of kinetic parameters resulted in a significant increase in V_max_ by 35%, but a similar value of K_Na_, when compared with the CW group (Figure 1b,c). When comparing the ZOD group with the CW group, the enzyme activity was 51% higher in the presence of 2 mmol∙L^−1^ of NaCl. With increasing NaCl concentration, the difference in the activity gradually decreased to 32%, observed in the presence of 100 mmol∙L^−1^ of NaCl (Figure 1a). The value of parameter V_max_ was increased by 29% with a statistically significant decrease in K_Na_ by 17% when compared with the CW group (Figure 1b,c).

Remarkable differences were also observed when comparing the ZDF fa/fa and ZL rats. The ZO and ZOD animals showed higher enzyme activity by approximately 20% throughout the investigated concentration range of NaCl (Figure 1a), resulting in higher V_max_ by 19% in the ZO group and by 14% in the ZOD group without statistically significant alterations of K_Na_ (Figure 1b,c).

A comparison of ZO and ZOD groups showed similar Na,K-ATPase activities when activating the enzyme with increasing NaCl concentrations without significant changes in kinetic properties of the enzyme (Figure 1b,c).

**Figure 2:**
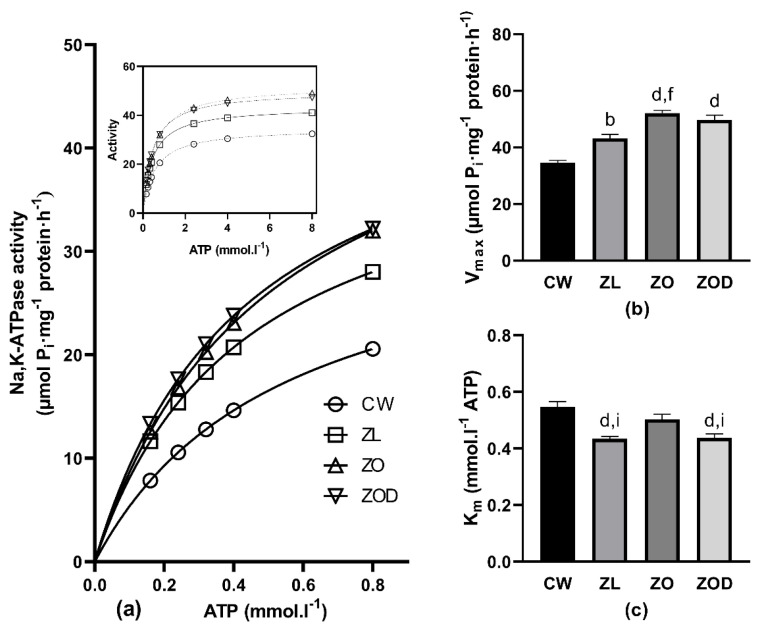
Activation of the Na,K-ATPase enzyme by substrate ATP in the renal cortex. (Panel **a**) Activation of the Na,K-ATPase by low concentrations of substrate ATP. Inset: activation of the enzyme in the whole investigated concentration range of ATP in Wistar rats (CW, *n* = 8), lean fa/+ (ZL, *n* = 8), and Zucker diabetic fatty (ZDF) fa/fa rats divided into ZO (ZDF fa/fa obese, glycemia < 10 mmol·L^−1^, *n* = 8) and ZOD (ZDF fa/fa obese diabetic, glycemia > 10 mmol·L^−1^, *n* = 8) groups. Error bars are not depicted, as they are shorter than the size of the symbols. (Panel **b**) V_max_ values in all experimental groups. (Panel **c**) K_m_ values in all experimental groups. The animals were sacrificed at the age of 38–39 weeks. Data represent means ± standard errors of mean. For statistical analyses, one-way analysis of variance with Tukey’s post hoc test was used: b: *p* < 0.01, d: *p* < 0.0001 vs. CW; f: *p* < 0.01 vs. ZL; i: *p* < 0.05 vs. ZO.

Activation of the renal Na,K-ATPase with increasing substrate concentrations resulted in higher enzyme activity throughout the investigated ATP concentration range in all Zucker rats (ZL, ZO, ZOD) compared with Wistar rats (Figure 2a). In the presence of the lowest studied concentration of ATP (0.16 mmol∙L^−1^), the increase of activity represented 48%, 60%, and 70% in the ZL, ZO, and ZOD groups, respectively. With increasing concentrations of substrate, the difference in the enzyme activity decreased stepwise. In the presence of the highest studied concentration of ATP (8.00 mmol∙L^−1^), the increase in activity represented 26%, 51%, and 46% of the ZL, ZO, and ZOD groups respectively. All Zucker rats (ZL, ZO, ZOD) also showed higher V_max_ values, independent of their health condition, compared with the CW group. In the ZL group, the increase represented 25%; in the ZO group, the increase was 50%, and in the ZOD group, it was 44% (Figure 2b,c). The values of K_m_ were significantly lower (by 20%) in ZL and ZOD groups, but without significant changes in the ZO group when compared with the CW group.

Comparing the ZDF fa/fa and ZL rats resulted in higher NaK-ATPase activities in both ZDF fa/fa rats—ZO and ZOD—at all applied concentrations of ATP (Figure 2a).

ZO animals showed the least difference in the enzyme activity in the presence of 0.24 mmol∙L^−1^ of ATP—approximately 9% lower in comparison with the ZL group. With increasing concentrations of substrate, the difference in enzyme activity increased stepwise to 20%, observed in the presence of 8.0 mmol∙L^−1^ of ATP (Figure 2a). Evaluation of kinetic parameters resulted in higher V_max_ by 21% and higher K_m_ by 16% in the ZO group when compared with the ZL group (Figure 2b,c). The ZOD animals showed higher enzyme activity by approximately 15% throughout the investigated concentration range of ATP (Figure 2a); however, V_max_ and K_m_ values were unchanged when compared with the ZL group (Figure 2b,c).

In ZDF fa/fa rats, a biphasic effect of their condition on Na,K-ATPase activity was observed. Below the concentration of 0.8 mmol∙L^−1^ of ATP, the enzyme activities were slightly lower in the ZO group than in the ZOD rats. On the other hand, an additional increase in substrate concentration was followed by decreased enzyme activities in the ZOD group when compared with the ZO group (Figure 2a). Evaluation of kinetic parameters showed similar V_max_ values in both groups, but the K_m_ value was significantly lower by 13% in the ZOD group when compared with the ZO group (Figure 2b,c).

## 4. Discussion

The use of animal models is a generally accepted approach to the analysis of T2DM and its complications. The missense mutation in the leptin receptor gene (fa/fa) in ZDF rats allows the study of T2DM in the condition of obesity. To ensure that the obtained research data will be interpreted correctly, a comprehensive characterization of the ZDF rat model is inevitable. The present study identified differences between two subgroups of ZDF obese rats (with well- and less developed T2DM) in renal properties, namely Na,K-ATPase enzyme kinetics and oxidative stress parameters. In the majority of experiments, ZDF obese fa/fa rats are compared with heterozygous lean fa/+ rats. A question may arise whether ZL rats are optimal controls to study all aspects of T2DM. Therefore, our additional aim was to compare lean fa/+ rats with independent control Wistar rats.

### 4.1. Basic Biometric and Biochemical Parameters

Analysis of body weight showed differences between both subgroups of ZDF fa/fa rats as well as both studied control groups. Although the ZDF rats with well-developed hyperglycemia (ZOD group) had higher BW than control ZL rats, the comparison with age-matched independent control Wistar rats showed no differences. Only ZDF rats with lower blood glucose levels (ZO group) exhibited higher BW, i.e., obesity (one of the basic characteristics of ZDF rats) in comparison with both controls: CW and ZL rats.

The differences in severity of T2DM were associated with differences in kidney weight—both normalized to BW and absolute. However, KW in both control groups did not differ after adjustment to BW.

Previous studies have shown a progressive, time-dependent development of diabetic complications in ZDF fa/fa rats. Rats with severe diabetes showed higher glucose levels when compared with age-matched mildly diabetic rats at 24 weeks of age [9]. Another comparison of older severely diabetic ZDF rats with mildly diabetic ones at the age of 30 weeks also showed significantly higher glucose levels [8]. Our data confirmed the age-dependent trend in diabetes severity by revealing increased glucose levels, namely by 176% in ZOD rats compared with the ZL control and by 107% in the ZOD group compared with obese mildly diabetic ZO animals at 36 weeks of life.

Comparison with Wistar rats showed significant differences in plasma insulin levels in rats aged 36 weeks, pointing out the importance of using two different control groups. Data obtained in all three Zucker rat groups (ZL, ZOD, and ZO) indicate significant changes in glucose metabolism. A study by Wang et al. showed significantly increased insulin levels in 24-week-old obese mildly diabetic Zucker rats in comparison with lean rats [9]. Our data on older rats confirmed this observation. Severe diabetes in obese ZDF rats revealed a significant decrease in insulin levels when compared with mildly diabetic animals in an age-dependent manner.

Regarding the plasma urea concentration, higher levels in ZDF rats have already been documented [23]. However, this study revealed that ZDF (fa/fa) rats characterized by lower glycemia (i.e., ZO group) did not differ from both control groups (CW and ZL). The concentration of urea in blood plasma was higher only in those ZDF (fa/fa) rats that were hypoinsulinemic and hyperglycemic, i.e., in ZOD rats. Notably, we also registered a significant difference between both controls, suggesting some degree of renal pathology in heterozygous (fa/+) animals that are commonly used as controls in experiments utilizing ZDF (fa/fa) rats.

### 4.2. Parameters of Oxidative and Carbonyl Stress

Even though the oxidative imbalance in the renal tissue of ZDF rats is well-documented, the current study used two novel approaches, which provides a deeper insight into the topic of oxidative stress in diabetes. One approach involved differentiating the ZDF fa/fa animals into two subgroups according to lower or higher blood glucose levels. The other approach focused on the distribution of oxidative as well as carbonyl stress symptoms in the renal cortex and medulla.

Our data on disrupted lipid components in the renal cortex of both ZDF (fa/fa) groups independent of diabetes severity are in line with previous observations [14,15,16] as indicated by increased concentrations of TBARS in our study.

The increase in antioxidant status estimated by TAC measurement in the renal cortex of ZO and ZOD groups may represent a defense mechanism against higher levels of oxidative stress in the condition of T2DM, as documented in the plasma of 1-year-old ZDF individuals [24]. However, this increase in TAC was not observed in the medullary tissue which, in the ZOD group, may be associated with TBARS levels even higher than in the renal cortex.

The unchanged levels of AGEs and fructosamine did not confirm the expected effect of increased plasma glucose levels on signs of carbonyl stress in the renal tissue. This resistance of the kidney against hyperglycemia seems to be organ-specific, as a significant increase in AGEs was documented in the left ventricle of the heart [10]. Notably, the level of AGEs in the renal cortex was highest in the ZL rats that exhibited the lowest body weight. It was shown that AGEs are lipophilic and, thus, are preferentially stored in the fat tissue [25]. In the case of lower body fat content (typical of ZL rats in our experiment), AGEs could be stored in higher amounts in other tissues, e.g., in the renal cortex, as suggested by this study. Paradoxically, the rats with the highest BW in the ZO group showed the lowest concentration of AGEs in the renal cortex—significantly lower in comparison with the ZL group.

### 4.3. Kinetic Measurements of Na,K-ATPase Enzyme

The present study provides new information concerning the molecular principles in the variability of Na,K-ATPase properties in ZDF rats as animal models of obesity and T2DM. The increase in Na,K-ATPase activities in all three investigated groups of Zucker rats (ZL, ZOD, and ZO), when compared with independent control Wistar rats, was probably caused by an increased number of active enzyme molecules in kidneys, as indicated by the higher value of V_max_ in both types of enzyme activation. A lowered K_Na_ value in obese diabetic ZDF rats (ZOD) indicates an improved ability of the enzyme to bind sodium. The ability to bind substrate ATP was also improved in ZL and ZOD rats, as indicated by the significant decrease in the K_m_ value when compared with Wistar control rats. Thus, our findings revealed important differences in the properties and functionality of Na,K-ATPase in the renal cortex, which may induce variations in the housekeeping of sodium balance in the investigated rat strains.

In ZDF fa/fa rats, obesity was accompanied by an increase in active enzyme molecules, as indicated by the higher V_max_ value when activating the enzyme with increased concentrations of substrate ATP or with sodium ions. This finding is consistent with previous observations documenting the involvement of renal Na,K-ATPase in the development of sodium retention in obesity and diabetes. The presence of the enzyme was higher in the kidney cortex of obese Zucker rats compared with the lean strain [26]. A similar increase in sodium retention as a consequence of higher expression and activity of Na,K-ATPase in the renal cortex was documented using a different rat model of hyperglycemia induced by chronic intraperitoneal glucose loading [27]. The ability to bind sodium was unchanged in obese animals, as shown by similar values of K_Na_ in lean rats as well as in obese mildly diabetic ZDF rats (ZO). However, the ability to bind ATP was lower, as indicated by the increased value of K_m_ when comparing the ZO and ZL groups. Thus, obese nondiabetic rats are likely to use the energy substrate less efficiently than lean rats.

It was shown previously that not all obese ZDF rats suffer from diabetes. Studies using Western blot revealed slightly lower Na,K-ATPase levels in the kidneys of obese diabetic rats when compared with obese nondiabetic rats [28]. Our analysis of this phenomenon showed a decrease in active enzyme molecules in severely diabetic obese ZOD rats when compared the mildly diabetic ZO group, as indicated by the V_max_ value. However, the difference was not statistically significant. This effect of diabetes was probably associated with the improved ability of the enzyme to utilize energy substrate, especially in the presence of lower physiologically relevant concentrations of ATP, as indicated by the lowered value of K_m_.

The link between the Na,K-ATPase activity and oxidative stress suggests that increased lipid peroxidation in the renal cortex of ZDF rats could negatively affect Na,K-ATPase activity. However, we have observed higher renal Na,K-ATPase activities in ZDF fa/fa rats than those found in control rats. Thus, in the present study, the increase in oxidative stress was not associated with an impairment of Na,K-ATPase functionality as suggested previously [17,18]. The transport of Na^+^ ions from the tubular cells is a prerequisite for the secondary active transport mechanisms involved in the reabsorption of multiple substances from the tubular fluid, including glucose. An increase in Na,K-ATPase activity in ZDF rats might be a consequence of increased glucose load in tubular fluid and higher demands for its reabsorption. It was shown that increased glucose levels were associated with increased Na,K-ATPase expression in proximal tubular epithelial cells [29].

## 5. Conclusions

The data obtained by our study allow us to perform three kinds of comparisons. First, the data allow us to evaluate the differences between lean fa/+ and Wistar rats. Second, we can compare the differences between control rat strains and ZDF fa/fa rats and, finally, between the ZDF fa/fa rats with well- and less developed T2DM.

Thiol–disulfide redox balance in the renal medulla and Na,K-ATPase properties in the renal cortex showed differences between both controls, lean fa/+ and Wistar rats, while the observed measurements in lean fa/+ rats showed deviation towards the values observed in ZDF fa/fa rats. The similar genetic background of lean fa/+ rats and ZDF fa/fa rats may render lean fa/+ rats, at least to some extent, predisposed to the anomalies observed in ZDF fa/fa rats.

ZDF fa/fa rats showed higher lipid peroxidation independent of diabetes severity consistently in the renal cortex, which was most probably compensated by an increase in total antioxidant capacity when compared with controls. In addition, ZDF fa/fa rats showed higher renal Na,K-ATPase activities than control rats in the wide range of ATP and sodium concentrations.

Regarding the differences within ZDF fa/fa animals depending on diabetes severity, we may conclude that well-developed T2DM (ZDF rats with higher glycemia and lower insulin concentration) is accompanied by increased lipid peroxidation in the renal medulla in comparison with ZDF rats with higher insulin levels and, thus, lower glucose concentrations in blood. However, ZDF fa/fa rats with well-developed T2DM showed a better ability of the Na,K-ATPase enzyme to utilize the ATP energy substrate.

## Figures and Tables

**Table 1:** General characteristics of the experimental animals.

Group	BW (g)	KW (g)	KW/BW × 10^3^	Glucose (mmol·L^−1^)	Insulin (ng·mL^−1^)
CW	494 ± 22	1.45 ± 0.04	2.78 ± 0.09	7.3 ± 0.1	7.9 ± 1.5
ZL	417 ± 8 ^b^	1.23 ± 0.03 ^c^	2.97 ± 0.05	6.7 ± 0.2	1.6 ± 0.08 ^b^
ZO	628 ± 8 ^d,h^	1.59 ± 0.05 ^h^	2.54 ± 0.05 ^f^	8.8 ± 0.2 ^e^	19.3 ± 2.1 ^d,h^
ZOD	528 ± 17 ^g,j^	1.77 ± 0.04 ^d,h,i^	3.36 ± 0.12 ^d,f,l^	18.3 ± 1.2 ^d,h,l^	8.7 ± 1.0 ^f,l^

Body weight (BW), kidney weight (KW), fasting blood glucose and plasma insulin level in Wistar rats (CW, *n* = 8), lean fa/+ (ZL, *n* = 8), and Zucker diabetic fatty (ZDF) fa/fa rats divided into ZO (ZDF fa/fa obese, glycemia < 10 mmol·L^−1^, *n* = 8) and ZOD (ZDF fa/fa obese diabetic, glycemia > 10 mmol·L^−1^, *n* = 8) groups. The animals were sacrificed at the age of 38–39 weeks. Data represent means ± standard errors of mean. For statistical analyses, one-way analysis of variance with Tukey’s post hoc test was used: b: *p* < 0.01, c: *p* < 0.001, d: *p* < 0.0001 vs. CW; e: *p* < 0.05, f: *p* < 0.01, g: *p* < 0.001, h: *p* < 0.0001 vs. ZL; i: *p* < 0.05, j: *p* < 0.01; l: *p* < 0.0001 vs. ZO.

**Table 2:** Parameters of oxidative stress and antioxidant status in the renal tissue.

Cortex					
Group	GSH/GSSG	AOPP (µmol·g^−1^)	TBARS (µmol·L^−1^)	TAC (mmol·L^−1^)	FRAP (mmol·L^−1^)
CW	2.9 ± 0.2	10.2 ± 0.8	2.1 ± 0.3	4.1 ± 0.1	0.63 ± 0.02
ZL	3.2 ± 0.2	13.2 ± 1.3	2.9 ± 0.4	4.3 ± 0.1	0.62 ± 0.02
ZO	2.7 ± 0.1	10.1 ± 0.3	6.2 ± 0.6 ^d,h^	5.1 ± 0.1 ^d,h^	0.55 ± 0.03
ZOD	3.0 ± 0.2	11.1 ± 1.1	4.7 ± 0.5 ^b,e^	5.3 ± 0.1 ^d,h^	0.58 ± 0.02
**Medulla**					
**Group**	**GSH/GSSG**	**AOPP** **(µmol·g^−1^)**	**TBARS** **(µmol·L^−1^)**	**TAC** **(mmol·L^−1^)**	**FRAP** **(mmol·L^−1^)**
CW	3.8 ± 0.4	9.0 ± 0.2	9.5 ± 0.9	4.3 ± 0.1	0.47 ± 0.02
ZL	6.6 ± 0.7 ^a^	10.0 ± 0.7	6.0 ± 0.6	4.4 ± 0.1	0.46 ± 0.03
ZO	6.5 ± 1.0	9.5 ± 1.4	6.7 ± 1.1	4.5 ± 0.1	0.51 ± 0.07
ZOD	6.4 ± 0.8	8.4 ± 0.3	11.3 ± 2.2 ^e^	4.5 ± 0.1	0.57 ± 0.04

GSH/GSSG—the ratio of reduced and oxidized glutathione; AOPP—advanced oxidation protein products; TBARS—thiobarbituric acid-reactive substances, TAC—total antioxidant capacity; FRAP—ferric reducing antioxidant power in Wistar rats (CW, *n* = 8), Zucker diabetic fatty (ZDF) lean fa/+ (ZL, *n* = 8), and ZDF fa/fa rats divided into ZO (ZDF fa/fa obese, glycemia < 10 mmol·L^−1^, *n* = 8) and ZOD (ZDF fa/fa obese diabetic, glycemia > 10 mmol·L^−1^, *n* = 8) groups. The animals were sacrificed at the age of 38–39 weeks. Data represent means ± standard errors of mean. For statistical analyses, one-way analysis of variance with Tukey’s post hoc test was used: a: *p* < 0.05, b: *p* < 0.01, d: *p* < 0.0001 vs. CW; e: *p* < 0.05; h: *p* < 0.0001 vs. ZL.

**Table 3:** Parameters of carbonyl stress in the renal tissue.

	Cortex		Medulla	
Group	Fructosamine (µmol·g^−1^)	AGEs (g·g^−1^)	Fructosamine (µmol·g^−1^)	AGEs (g·g^−1^)
CW	111 ± 5	0.15 ± 0.02	89 ± 7	0.13 ± 0.01
ZL	122 ± 9	0.21 ± 0.02	84 ± 5	0.17 ± 0.02
ZO	102 ± 6	0.13 ± 0.01 ^e^	94 ± 6	0.15 ± 0.02
ZOD	115 ± 9	0.15 ± 0.01	101 ± 8	0.14 ± 0.01

Wistar rats (CW, *n* = 8), lean fa/+ (ZL, *n* = 8) and Zucker diabetic fatty (ZDF) fa/fa rats divided into ZO (ZDF fa/fa obese, glycemia < 10 mmol·L^−1^, *n* = 8) and ZOD (ZDF fa/fa obese diabetic, glycemia > 10 mmol·L^−1^, *n* = 8) groups. AGEs—advanced glycation end products. The animals were sacrificed at the age of 38–39 weeks. Data represent means ± standard errors of mean. For statistical analyses, one-way analysis of variance with Tukey’s post hoc test was used: e: *p* < 0.05 vs. ZL.

## Data Availability

The data that support the findings of this study are available in this article or from the corresponding author upon reasonable request.
